# Patterns, trends, and factors associated with contraceptive use among adolescent girls in Zambia (1996 to 2014): a multilevel analysis

**DOI:** 10.1186/s12905-020-01050-1

**Published:** 2020-08-26

**Authors:** Mumbi Chola, Khumbulani Hlongwana, Themba G. Ginindza

**Affiliations:** 1 Department of Public Health Medicine, School of Nursing and Public Health, University of KwaZulu Natal, Durban, South Africa; 2 Department of Epidemiology & Biostatistics, School of Public Health, University of Zambia, Lusaka, Zambia

**Keywords:** Adolescent girls, Contraceptive use, Patterns and trends, Multilevel analysis, Zambia

## Abstract

**Background:**

Despite high levels of pregnancy and childbearing among adolescents in Africa, contraceptive use remains low. Examining variations in contraceptive use among adolescent girls is vital for informing programs to improve contraceptive utilisation among this segment of the population. This study aimed to examine the patterns, trends, and factors associated with contraceptive use among adolescents in Zambia over the period 1996–2014.

**Methods:**

The study involved an analysis of data from 1996, 2001/2, 2007 and 2013/14 Zambia Demographic and Health Surveys focusing on adolescent girls aged 15–19 years. Analysis entailed descriptive statistics and estimation of multilevel logistic regression models examining variations in contraceptive use among adolescent girls over time. Estimates with *p*-values less than 0.05 were considered statistically significant.

**Results:**

Results showed that contraceptive use remains low and ranged from 7.6% in 1996 to 10.9% in 2013/14, reflecting a change of 3.3 percentage points over 18 years. Over the 18 years, contraceptive use was significantly associated with age, level of education, and marital status. Older adolescent girls and those with higher levels of education were significantly more likely to use contraception compared to younger ones and those with lower levels of education. Although initially significant (AOR 0.556, 95% CI 0.317, 0.974 in 1996), rural-urban differences disappeared between 2001/2 and 2007 but re-emerged in 2013/14 (AOR 0.654, 95% CI 0.499, 0.859). Across all survey years, adolescents who were married or living with a partner were significantly more likely to use contraceptives compared to those who were not married.

**Conclusions:**

The findings suggest the need for targeted interventions to improve contraceptive use among sexually active adolescent girls in the country in general, and those who are disadvantaged in particular.

## Background

The World Health Organisation (WHO) estimated that every year, approximately 21 million girls between the ages of 15 and 19 years, and 2 million girls aged below 15 years become pregnant in developing regions, with an estimated 16 million girls between ages 15 and 19 years and 2.5 million girls below the age 16 years giving birth [1]. Approximately half of these pregnancies (49%), reported among adolescents between ages 15–19 who live in low-income regions, are unintended, and over 50% result in abortions, usually under unsafe conditions [2]. WHO further states that although the global birth rate among adolescents reduced to 47 births per 1000 women in 2015 from 65 births per 1000 women in 1990, the adolescent population continues to grow globally and adolescent pregnancies are also projected to increase by 2030, with West and Central Africa and Eastern and Southern Africa experiencing the most substantial increases [1]. Projections also show that the number of adolescent mothers will reach a high of 86 million by 2030 [3].

Pregnancies among adolescent girls have serious consequences which can significantly affect the lives of the adolescent mothers and those of the children. Early and unintended pregnancies among adolescents have been associated with adverse health, educational, social, and economic outcomes [4]. The impact on adolescent mothers includes risks of maternal death, illness, and disability such as obstetric fistula, complications of unsafe abortion, sexually transmitted infections; including HIV, and health risks to infants [5]. About 70,000 adolescent girls in low-income countries (LICs) die annually of causes related to pregnancy and childbirth [5]. Among adolescent girls aged 15–19 years in LICs, pregnancy and childbirth complications are the second leading cause of death, and babies born to these mothers have increased health risks compared to those born to older women [2]. Early and unintended pregnancy disrupts adolescent girls’ schooling, thus affecting their future economic opportunities, including reducing job market opportunities [5, 6]. The effects of adolescent childbearing also extend to the health of babies with higher perinatal deaths and low birth weight among children born to mothers aged below 20 years [5–7].

Despite the high rates of pregnancies and births among adolescents, contraceptive use among this segment of the population remains low globally, particularly in LICs, such as those in Africa [8–10]. Evidence shows that contraceptive prevalence rate (CPR) among adolescent females aged 15–19 years in LICs is 21% for all methods (modern and traditional) [8, 11, 12]. The low use of contraception among adolescents occurs against the backdrop of evidence that using family planning methods has benefits that could reduce some of the negative consequences of adolescent pregnancy and childbearing. These benefits include the freedom to decide how many children to have and child spacing, improvements in health-related outcomes, such as a reduction in maternal mortality and infant mortality [13–15] and improvements in schooling and economic outcomes [16, 17].

Studies show that various factors influence adolescents’ decision making regarding whether or not to use contraceptives. These include individual; family, societal or peer; health system; and cultural and religious factors. This paper focuses on individual-level factors, which include education [10, 18, 19], knowledge of contraceptives [20, 21], fear, shame, myths and stigma [22, 23] and fear of side effects and adverse reactions [24, 25].

In Zambia, contraceptive use among adolescents remains low despite the evidence showing almost universal knowledge of at least one modern contraceptive method [26]. Statistics show that contraceptive prevalence among women 15–49 years has been increasing over time in the country. Estimates, for instance, show that use of any method among currently married women increased from 15.2% in 1992 to 49.0% in 2013–14 while use of modern methods increased from 8.9 to 44.8% over the same period [26]. Among adolescents, contraceptive use remains low with only 10.2% using any modern contraceptive method in 2013/14 [26]. There is limited understanding of the patterns and trends of contraceptive use among adolescents and associated individual-level factors and how these have changed over the past two decades. The aim of this study was to examine the patterns, trends, and factors associated with contraceptive use among adolescents in Zambia over the period 1996 to 2014.

## Methods

This study involved an analysis of cross-sectional data from four Zambia Demographic and Health Surveys (1996, 2001/2, 2007 and 2013/14). The Zambia Demographic and Health Survey (ZDHS) is a nationally representative sample survey of Zambian households. The main objective of the ZDHS is to provide information on fertility levels and trends, mortality, family planning, as well as indicators on maternal and child health including HIV/AIDS [26]. The sample for the ZDHS is designed to provide estimates of population and health indicators at the national and provincial levels [26]. The sample design allows for specific indicators, such as contraceptive use, to be calculated for all provinces in Zambia. The sampling frame used for the ZDHS is usually adopted from the Census of Population and Housing of the Republic of Zambia (CPH) provided by the Central Statistical Office.

A representative sample of households was drawn for all the ZDHS surveys using a two-stage stratified cluster sample design, with Enumeration Areas (EAs) (or clusters) selected during the first stage and households selected during the second stage. The sample was stratified in two stages from the CPH frames (1990, 2000 and 2010). Stratification was achieved by dividing every province into urban and rural areas. Provinces were stratified into 18 strata in earlier surveys and 20 strata in the 2013/14 survey. Samples were selected independently in every stratum through a two-stage selection process. Stratification and proportional allocation were achieved, at all lower geographical/administrative levels, by sorting the sampling frame according to the geographical/administrative order and using a probability proportional to size selection process in the first stage. All women and men aged, 15–49 and 15–59 respectively, who were either permanent residents of the households in the sample or were visitors present in the household on the night before the survey, were eligible for interview [26].

The target population in this study included adolescent girls aged 15–19 years captured in the ZDHS surveys. All those who responded to the question on current contraceptive method were included in the analysis. Current use of a contraceptive method, which was the dependent variable, was recoded into a binary outcome, those currently using and those not using any method. Explanatory variables included in the analysis were age, type of place of residence, province, highest level of education, marital status at the time of interview, literacy, and knowledge of any contraceptive method (Table 1). Since the ZDHS involved more than one level of stratification, analysis took into account cluster and household variables. Adolescents who had never had sex were excluded from the analysis.

**Table 1:** Definition of variables

Variable	Definition and measurement
Current Contraceptive Use	Contraception use: 0 = Not using a method; 1 = Using a method
Knowledge of Any Method	Knowledge of any modern contraceptive method: 0 = Knows no method; 1 = Knows modern methods”
Age	Respondent’s current age in competed years: ranges from 15 to 19
Current Marital Status	Marital status of the respondent: 0 = Never in Union; 1 = Married/Living with Partner; 2 = Widowed/Separated/Divorced”
Residence	Type of place of residence: 0 = Urban; 1 = Rural
Province	Province or region of residence: 1 = Central; 2 = Copperbelt; 3 = Eastern; 4 = Luapula; 5 = Lusaka; 6 = Muchinga; 7 = Northern; 8 = North Western; 9 = Southern; 10 = Western
Highest Educational Level	Highest level of education attained: 0 = No Education; 1 = Primary; 2 = Secondary & Higher
Literacy	Whether a respondent who attended primary schooling can read a whole or part of a sentence: 0 = Cannot read at all; 1 = Able to read only parts of sentence; 2 = Able to read whole sentence
Currently working	Whether the respondent was currently working (at the time of the survey): 0 = No; 1 = Yes

Data from the four ZDHS surveys were combined by appending the data sets from 1996, 2001/2 and 2007 to the 2013/14 ZDHS. We started by conducting descriptive analysis and presented the results as proportions. Where appropriate, Chi-square and Fischer’s exact tests were used to test for the significance of association between the outcome and explanatory variables. We then conducted multilevel multivariate logistic regression analysis – factoring in random effects–to determine the predictors of contraceptive use. Analysis was conducted using Stata 15/MP (StataCorp LLC) and estimates with *p*-value less than 0.05 were considered statistically significant.

## Results

### Sample characteristics

A total of 9072 adolescent girls were included in the analysis across the four ZDHS surveys. The majority (41%) were from the 2013/14 ZDHS while 18% were from the 2007 survey, 20% from the 2001/2 survey and 22% from the 1996 survey. Table 2 shows the distribution of adolescent girls included in the analysis by background characteristics across the survey years.

**Table 2:** Distribution of adolescent girls by background characteristics and survey year, ZDHS 1996–2013/14

Variable	ZDHS 96	ZDHS 2001/2	ZDHS 2007	ZDHS 2013/14	Total
	**N** = 1982	**N** = 1806	**N** = 1598	**N** = 2686	**N** = 9072
**Current Contraceptive Use**					
No	1831 (92.4%)	1630 (90.3%)	1424 (89.1%)	3296 (89.4%)	8181 (90.2%)
Yes	151 (7.6%)	176 (9.8%)	174 (10.6%)	390 (10.9%)	891 (9.8%)
**Knowledge of Any Modern Method**					
Knows no method	247 (12.5%)	127 (7.0%)	150 (9.4%)	162 (4.4%)	686 (7.6%)
Knows modern method	1728 (87.5%)	1677 (93.0%)	1447 (90.6%)	3519 (95.6%)	8371 (92.4%)
**Age**					
15 Years	395 (19.9%)	372 (20.6%)	370 (23.2%)	735 (19.9%)	1872 (20.6%)
16 Years	419 (21.1%)	326 (18.1%)	330 (20.7%)	759 (20.6%)	1834 (20.2%)
17 Years	369 (18.6%)	325 (18.0%)	303 (19.0%)	674 (18.3%)	1671 (18.8%)
18 Years	404 (20.4%)	406 (22.5%)	299 (18.7%)	774 (21.0%)	1883 (20.8%)
19 Years	395 (19.9%)	377 (20.9%)	296 (18.5%)	744 (20.2%)	1812 (20.0%)
**Current Marital Status**					
Never Married	1435 (74.2%)	1307 (72.4%)	1302 (81.5%)	3058 (83.0%)	7102 (78.3%)
Married/Living with Partner	501 (25.3%)	449 (24.9%)	270 (16.9%)	572 (15.5%)	1792 (19.8%)
Widowed/Separated/Divorced	46 (2.3%)	50 (2.8%)	26 (1.6%)	56 (1.5%)	178 (2.0%)
**Residence**					
Urban	799 (40.3%)	639 (35.4%)	809 (50.6%)	1850 (50.2%)	4097 (45.2%)
Rural	1183 (59.7%)	1167 (64.6%)	789 (49.4%)	1836 (49.8%)	4975 (454.8%)
**Province**					
Central	170 (8.6%)	223 (12.4%)	152 (9.5%)	327 (8.9%)	872 (9.6%)
Copperbelt	309 (15.6%)	235 (13.0%)	190 (11.9%)	417 (11.3%)	1151 (12.7%)
Eastern	244 (12.3%)	192 (10.6%)	196 (12.3%)	472 (12.8%)	1104 (12.2%)
Luapula	242 (12.2%)	159 (8.8%)	152 (9.5%)	279 (7.6%)	832 (9.2%)
Lusaka	274 (13.8%)	204 (11.3%)	224 (14.0%)	434 (11.8%)	1136 (12.5%)
Muchinga^a^	–	–	–	340 (9.2%)	1001 (11.0%)
Northern	204 (10.3%)	288 (16.0%)	169 (10.6%)	352 (9.6%)	821 (9.1%)
North western	114 (5.8%)	198 (11.0%)	157 (9.8%)	381 (10.3%)	943 (10.4%)
Southern	205 (10.3%)	166 (9.2%)	191 (12.0%)	377 (10.2%)	905 (10.0%)
Western	220 (11.1%)	141 (7.8%)	167 (10.5%)	307 (8.3%)	307 (3.4%)
**Highest Educational Level**					
No education	174 (8.8%)	158 (8.8%)	69 (4.3%)	69 (1.9%)	470 (5.2%)
Primary	1254 (63.3%)	1073 (59.5%)	756 (47.5%)	1418 (38.6%)	4501 (49.8%)
Secondary	553 (27.9%)	574 (31.8%)	767 (48.2%)	2183 (59.5%)	4077 (45.0%)
**Literacy**^b^					
Cannot Read at All	–	765 (42.8%)	386 (24.6%)	750 (20.5%)	1901 (27.1%)
Able to Read Only Parts of Sentence	–	188 (10.5%)	151 (9.6%)	283 (7.7%)	622 (8.9%)
Able to Read Whole Sentence	–	834 (46.7%)	1032 (64.8%)	2621 (71.7%)	4487 64.0%)

^*a*^ *- Data on Muchinga province not available for 1996, 2001/2 and 2007 surveys. Muchinga only became a province in 2011*

^*b*^ *- Data on literacy was not collected in the 1996 ZDHS*

Overall, contraceptive use over the 18 years has been low, with only 9.8% of adolescent girls aged between 15- and 19-years using contraceptives (Table 2). This ranged from 7.6% in 1996 to 10.9% in 2013/14, reflecting a 3.3 percentage point change over 18 years (*p* < 0.01). The distribution of respondents across ages ranged from 18.1 to 23.2% over the survey years. Regarding marital status, the proportion of adolescents who reported being married declined from 24.8% in 1996 to 15.1% in 2013/14 (Table 2). However, 19.3% of adolescent girls interviewed over the 18-year period from 1996 to 2014 were married at the time of the survey. Rural and urban distribution showed that in 1996 and 2001/2, the majority of the respondents were from rural areas (59.7 and 64.6% respectively). However, in 2007 and 2013/14, the distribution was almost even (50.6% vs 49.4% in 2007 and 50.2% vs 49.8% in 2013/14). The provincial distribution shows that overall, Copperbelt (12.7%), Lusaka (12.5%) and Eastern (12.2%) provinces had the highest proportion of adolescent girls; with Western province recording the lowest proportion of 3.4%.

There were also changes in the distribution of adolescent girls by highest level of education. In 1996 and 2001/2, the majority of respondents had attained primary school level education (63.3 and 59.4% respectively) while in 2007 and 2013/14, the majority had attained secondary level education (48.2 and 59.3% respectively; Table 2). Knowledge of modern contraceptive methods also increased over the reference period. In 1996, 87.7% of adolescents reported knowing modern contraceptive methods, which increased to 95.5% in 2013/14. Concerning literacy, the proportion of adolescents who could not read at all declined from 42.4% in 2001/2 to 20.4% in 2013/14. In contrast, the proportion of adolescents who could read whole sentences increased from 46.2% in 2001/2 to 71.1% in 2013/14. No data on literacy was collected in 1996.

### Patterns and trends in contraceptive use

Table 3 below describes the proportions of adolescent girls aged 15–19 years who used contraceptives across the survey years.

**Table 3:** Distribution of adolescent girls aged 15–19 years using contraception by background characteristics, 1996–2013/14

Current Contraceptive Use	1996		2001/2		2007		2013/14	
	%	N	%	N	%	N	%	N
**Marital Status**								
Never in union	4.3	1435	4.7	1307	6.8	1302	5.6	3058
Married/Living with Partner	17.0	416	24.3	449	28.5	270	36.4	572
Widowed/Separated/Divorced	8.7	46	10.0	50	34.6	26	19.6	56
*P*-Value	0.000		0.000		0.000		0.000	
**Province**								
Central	5.3	170	5.4	223	6.6	152	9.8	327
Copperbelt	6.5	309	11.1	235	6.3	190	6.7	417
Eastern	9.0	244	7.3	192	13.8	196	11.0	472
Luapula	2.1	242	13.2	159	3.3	152	5.7	279
Lusaka	8.0	274	15.7	204	8.5	224	12.7	434
Muchinga¶	-	-	-	-	-	-	7.9	340
Northern	9.3	204	7.6	288	7.1	169	9.1	352
North western	20.2	114	11.1	198	15.3	157	12.9	381
Southern	4.9	205	8.4	166	8.9	191	13.5	377
Western	9.6	220	9.2	141	28.7	167	15.6	307
*P*-Value	0.000		0.014		0.000		0.000	
**Residence**								
Urban	8.1	799	11.4	639	10.0	809	10.1	1850
Rural	7.3	1183	8.8	1167	11.8	789	11.1	1836
*P*-Value	0.476		0.075		0.255		0.297	
**Highest Education Level**								
Education	5.8	174	10.1	158	10.1	69	7.3	69
Primary	7.4	1254	9.2	1073	13.0	756	11.6	1428
Secondary & Higher	8.7	554	10.6	575	8.9	773	10.0	2197
*P*-Value	0.407		0.656		0.040		0.199	
Literacy*								
Cannot read at all	–		9.9	765	15.0	386	12.5	750
Able to read only par	–		11.2	188	9.9	151	9.2	283
Able to read whole se	–		9.1	834	9.7	1032	10.1	2621
*P*-Value			0.657		0.015		0.121	

*¶ – Data on Muchinga province not available for 1996, 2001/2 and 2007 surveys. Muchinga only became a province in 2011.*

* – Data on literacy was not collected in the 1996 ZDHS

#### Age

**Fig. 1:**
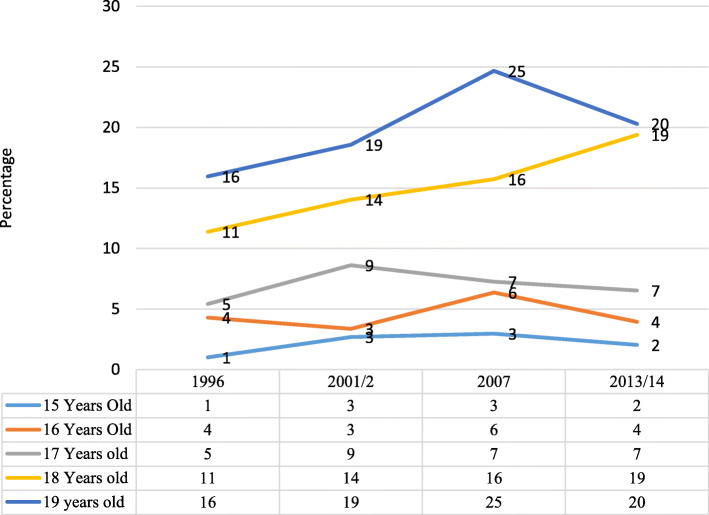
Trends in Contraceptive Use by Age over the period 1999 to 2013/14

Over the period 1996 to 2013/14, contraceptive use increased significantly with age (*p* = 0.000). The proportion using contraception was highest among 19-year old and lowest among 15-year old adolescent girls across survey years (Fig. 1). However, the proportion of 19-year old adolescent girls using contraception declined from 25% in 2007 to 20% in 2013/14.

#### Marital status

Differences in contraceptive use according to marital status across the four surveys were also statistically significant (*p* = 0.000). Adolescent girls who were married or living with a partner comprised the highest proportion of contraceptive users in 1996 (17.0%) and 2001/2 (24.3%). From 2001/2 to 2007, the proportion of contraceptive users increased across all groups with the most increases being among those married or living with a partner (24.3% in 2001/2 to 28.5% in 2007) and those who were widowed/ separated or divorced (10.0% in 2001/2 to 34.6% in 2007). By 2013/14, those who were married/living with a partner comprised the highest proportion of contraceptive users (36.4%).

#### Province

Variations in contraceptive use among adolescent girls by province show that in 1996, North-Western province had the highest proportion of users (20%) while Luapula province had the lowest (2%; Table 3). In the 2001/2 survey, Lusaka had the highest while Central province had the lowest proportion of adolescent girls using contraception (16 and 5%, respectively; Table 3). In 2007 and 2013/14, the proportion of adolescent girls using contraception was highest in Western province (29 and 16%, respectively) and lowest in Luapula province (3 and 6%, respectively). The observed differences in contraceptive use by province were statistically significant (*p* = 0.000).

#### Residence

Contraceptive use by urban-rural residence showed some variation over the period 1996–2013/14. Contraceptive use among urban adolescent girls increased between 1996 to 2001/2 from 8.1 to 11.4% before slightly declining to 10.1% in 2007 and remaining unchanged thereafter (Table 3). Among adolescent girls in rural areas, the proportion using contraception increased between 1996 and 2007 from 7.3 to 11.8% and remained largely unchanged thereafter.

#### Highest education level

The proportion of adolescent girls using contraception was generally low across all levels of education. In 2007 and 2013/14, use was highest among those with primary level education (13 and 12%, respectively), having increased from 7% in 1996 (Table 3). Among those with no education, only 7% reported using a contraceptive method in 2013/14, which was lower than the proportion using in 2001/2 and 2007 (10% in each case; Table 3). Among those with secondary level education, the proportion ranged between 9 and 11% in the period between 1996 and 2013/14 with 10% reporting currently using contraceptives in 2013/14.

### Determinants of contraceptive use

The odds of using contraceptives increased with age across all years. The odds of contraceptive use were higher among older adolescents than the younger ones across all survey years. However, significant differences were observed in 1996, 2007 and 2013/14. In 1996, adolescent girls aged 19 years were 4 times significantly more likely to use contraception compared to those aged 15 years (AOR 4.175, 95% CI 1.377, 12.656) while in 2007, they were twice as likely to use a method as their 15-year-old counterparts (AOR 2.667, 95% CI 1.255, 5.668) (Table 4). Results in Table 4 showed that in 2013/14, 18-year olds had the highest odds of contraceptive use compared to 15-year olds (AOR 2.717, 95% CI 1.496, 4.935.

**Table 4:** Odds ratios from multilevel logistic regression analysis examining variations in current use of contraception among adolescent girls aged 15–19 years in Zambia, ZDHS 1996–2013/14

	1996 (***n*** = 1181)				2001/2 (***n*** = 1042)				2007 (***n*** = 795)				2013/14 (***n*** = 1879)			
Current Contraceptive Use	AOR	*p*-values	[95%	CI]	AOR	*p*-values	[95%	CI]	AOR	*p*-values	[95%	CI]	AOR	*p*-values	[95%	CI]
**Age**																
15	1.000				1.000				1.000				1.000			
16	2.252	0.171	(0.705	7.194)	0.494	0.139	(0.195	1.256)	1.063	0.885	(0.464	2.435)	1.049	0.889	(0.534	2.063)
17	1.733	0.352	(0.544	5.521)	0.768	0.524	(0.342	1.728)	0.762	0.516	(0.335	1.732)	1.031	0.927	(0.539	1.970)
18	2.872	0.062	(0.947	8.706)	1.058	0.884	(0.494	2.265)	1.425	0.372	(0.655	3.101)	2.717	0.001	(1.496	4.935)
19	4.175	0.012	(1.377	12.656)	1.291	0.511	(0.603	2.764)	2.667	0.011	(1.255	5.668)	1.825	0.050	(1.001	3.328)
**Province**																
Central	1.000				1.000				1.000				1.000			
Copperbelt	1.345	0.544	(0.516	3.504)	2.671	0.017	(1.193	5.978)	1.262	0.640	(0.477	3.341)	0.904	0.750	(0.484	1.687)
Eastern	3.147	0.017	(1.230	8.052)	1.425	0.425	(0.596	3.406)	2.257	0.062	(0.959	5.311)	0.937	0.817	(0.542	1.620)
Luapula	0.539	0.319	(0.159	1.819)	3.234	0.004	(1.445	7.238)	0.390	0.117	(0.120	1.265)	0.602	0.161	(0.296	1.224)
Lusaka	1.705	0.273	(0.657	4.427)	3.731	0.001	(1.671	8.332)	1.280	0.591	(0.521	3.143)	1.302	0.354	(0.745	2.273)
Muchinga													0.793	0.472	(0.421	1.493)
Northern	2.871	0.030	(1.109	7.437)	1.559	0.257	(0.724	3.359)	0.976	0.961	(0.375	2.541)	0.703	0.253	(0.384	1.287)
North western	6.043	0.000	(2.301	15.875)	2.274	0.039	(1.041	4.967)	2.408	0.050	(1.000	5.800)	1.033	0.908	(0.592	1.803)
Southern	1.302	0.619	(0.460	3.688)	1.763	0.189	(0.756	4.110)	1.108	0.822	(0.453	2.713)	1.110	0.719	(0.630	1.954)
Western	2.301	0.080	(0.905	5.853)	1.735	0.208	(0.736	4.093)	5.967	0.000	(2.598	13.706)	1.406	0.238	(0.799	2.475)
**Residence**																
Urban	1.000				1.000				1.000				1.000			
Rural	0.556	0.040	(0.317	0.974)	0.827	0.422	(0.520	1.315)	0.658	0.051	(0.432	1.002)	0.654	0.002	(0.499	0.859)
**Highest Level of Education**																
No Education	1.000				1.000				1.000				1.000			
Primary	2.057	0.061	(0.968	4.371)	1.195	0.562	(0.655	2.179)	3.525	0.008	(1.386	8.964)	2.928	0.032	(1.099	7.801)
Secondary	3.080	0.009	(1.322	7.175)	2.330	0.015	(1.179	4.606)	3.389	0.015	(1.263	9.092)	3.584	0.011	(1.333	9.636)
**Current Marital Status**																
Never in union	1.000				1.000				1.000				1.000			
Married/Living with Partner	1.934	0.003	(1.258	2.975)	2.867	0.000	(1.928	4.263)	2.408	0.000	(1.562	3.712)	4.131	0.000	(3.071	5.559)
Widowed/Separated/Divorced	0.708	0.551	(0.227	2.207)	0.837	0.727	(0.309	2.271)	3.199	0.016	(1.247	8.208)	1.423	0.351	(0.678	2.984)
**Currently Working**																
No	1.000				1.000				1.000				1.000			
Yes	1.614	0.023	(1.070	2.436)	1.276	0.243	(0.847	1.921)	1.392	0.127	(0.911	2.127)	1.345	0.037	(1.018	1.777)
sigma_u	0.565		(0.261	1.223)	0.004		(0.000	120,604.000)	0.005		(3.04E-16	8.78E-10)	0.281		(0.051	1.538)
rho	0.088		(0.020	0.313)	0.000		(0.000	1.000)	8.12E-06		(2.81E-32	1.000)	0.023		(0.001	0.418)

Results from correlation analysis showed that although there were some positive and negative correlations between the factors considered in the analysis, these were not very strong (Table 5).

**Table 5:** Results from Collinearity Test

	Current Contraceptive Use	Age	Province	Residence	Highest Level of Education	Current Marital Status	Currently Working
Current Contraceptive Use	1						
Age	0.222	1					
Province	0.065	0.020	1				
Residence	−0.003	−0.011	0.124	1			
Highest Level of Education	0.001	0.091	−0.016	−0.361	1		
Current Marital Status	0.257	0.353	−0.026	0.144	−0.25	1	
Currently Working	0.099	0.134	0.103	0.216	−0.202	0.177	1

There were also disparities by province. In 1996, adolescent girls in North Western (AOR 6.043, 95% CI 2.301, 15.875), Eastern (AOR 3.147, 95% CI 1.230, 8.052), and Northern (AOR 2.871, 95% CI 1.109, 7.437) provinces reported the highest odds of using contraceptives compared to those in Central. In 2001/2, there were significant variations between Central province and Copperbelt (AOR 2.671, 95% CI 1.193, 5.978), Luapula (AOR 3.234, 95% CI 1.445, 7.238), Lusaka (AOR 3.731, 95% CI 1.671, 8.332) and North Western (AOR 2.274, 95% CI 1.041, 4.967) provinces (Table 4). In 2007, there were significant differences between Central and North Western and Western provinces. Adolescent girls in North Western and Western provinces were, respectively, 2.4 times and almost 6 times more likely to use contraceptives compared to those in Central province. There were no significant variations in contraceptive use by province in 2013/14.

Adolescent girls in rural areas were also less likely to use contraceptives compared with their urban counterparts. There were statistically significant variations in contraceptive use by place of residence in 1996 (AOR 0.556, 95% CI 0.317, 0.974) and 2013/14 (AOR 0.654, 95% CI 0.499, 0.859) (Table 4). Adolescent girls with secondary or higher levels of education were significantly more likely to use contraception compared to those with no education across all survey years. From 2007 onwards, adolescent girls with primary level education, like those with secondary level education, were also significantly more likely to use contraceptives compared to those with no education. Similarly, adolescent girls who were married or living with a partner were significantly more likely to use contraception than their never married counterparts across all survey years. In 2007, adolescent girls who were married or living with a partner, or were widowed, separated, or divorced were significantly more likely to use contraception than their never married counterparts.

In summary, the results show that adolescent girls who were most likely to use contraceptives were aged 18–19 years old, had secondary education or higher, and were either married or living with their partner. However, whereas initial significant urban-rural differences in contraceptive use among adolescent girls seemed to have been bridged between 2001/2 and 2007, such differences emerged in 2013/14.

Random-effects results from the multilevel model show that in 1996, 9% of variability in contraceptive use among adolescent girls was explained by inter-cluster variations while in 2013/14, inter-cluster variability explained only 2% of the variations. This shows that over time, variations in contraceptive use among adolescent girls across different geographic clusters were becoming less important compared to individual-level variations.

## Discussion

Contraceptive use among adolescent girls in Zambia remained low over the period 1996–2014 although knowledge of at least one modern method is almost universal. Results from this study showed that in recent years, age, education, residence, marital status, and working status were significantly associated with contraceptive use among adolescent girls. Significant rural-urban differences, which occurred in early years but disappeared in subsequent years, re-emerged in recent years while provincial disparities were bridged. In addition, the odds of using contraceptives increased as adolescent girls grew older and achieved higher levels of education. Furthermore, adolescent girls who were married or living with their partners were significantly more likely to use contraceptives compared with those who were never married.

The low contraceptive use among adolescents could be due to challenges with access to contraception and the actual use of methods. Health system issues, such as lack of adolescent-friendly health services, as well as health care worker attitudes [22], can deter adolescents from accessing contraceptives, particularly from health centres. Lack of access to contraceptive and family planning services at heath facilities affects the kind of information that adolescents have. Studies have shown that in addition to low contraceptive use and limited access to information and services, adolescents have poor knowledge of family planning [27]. Adolescents’ primary source of information was usually their peers, and the information received from such sources was mostly untrustworthy and distorted [28], thus perpetuating myths and misconceptions about contraception. The low use of contraception among adolescents could therefore be partly due to limited access to the right information and services.

Over the period 1996–2014, contraceptive use marginally increased with age. Adolescent girls aged 19 years were more likely to be using contraceptives at the time of the survey compared to 15-year-olds. This finding is consistent with those from other studies [8, 29]. This could be attributed to older female adolescents being more mature and knowledgeable about contraception and the importance of contraceptive use, unlike younger female adolescents. Furthermore, older female adolescents are more likely to be married, have higher education levels, and more likely to be active sexually compared to younger adolescents [30]. Adolescents who reported being married or living together with a man, or were separated, widowed, or divorced were more likely to use contraceptives compared to those who had never married. In recent surveys, adolescent girls who were separated, widowed, or divorced were also more likely to be using contraceptives compared to those who had never married. The findings are in line with those from other studies [10, 29, 30]. One study found that adolescent girls who were married or living with a man were significantly more likely to use contraception compared to those who were not married or living together with a partner [10]. Another study found that married adolescent girls had the highest odds of using contraceptives compared to those that never married or were formerly married [30, 31]. This could be due to partner support in using contraceptives among married women [32]. Married female adolescents may likely use contraceptives because they can afford more effective contraceptives than their unmarried counterparts due to financial support from the partner [10]. They are also more likely to practice family planning and take measures to prevent pregnancy compared to those who are not married due to regular exposure to sexual intercourse and the risk of unintended pregnancy.

The findings of this paper show that current contraceptive use increased with the level of education. Other studies have reported similar findings. Evidence shows that education affects contraceptive use among adolescents [18]. A study in Ghana found that the level of education was a significant factor in contraceptive use among women of reproductive age [33]. As the level of education increased, there was an increase in contraceptive use [10, 19]. This increase was more so in urban areas where urban adolescents, who typically have higher education, report a higher likelihood of contraceptive use, particularly condoms [21]. Adolescents who are more educated are more likely to appreciate the advantage of having fewer children and how this can positively impact their own economic productivity and the well-being of their children [33, 34].

The results reported in this study have implications for both policy and public health in general. The increased likelihood of contraceptive use among adolescent girls with higher levels of education suggests that keeping girls longer in school is likely to improve their reproductive health outcomes. In addition, the introduction of comprehensive sexuality education in primary school curriculum in Zambia is vital for providing comprehensive and age-appropriate information to adolescents who are at a pivotal stage of their lives. However, it is vital to also target adolescents who are outside the school system. Community-based activities, in addition to youth-friendly spaces in health centres, are essential to ensure correct information is provided to adolescents.

Furthermore, access to contraceptives is another issue that needs to be addressed. Information centres, such as schools, can also serve as distribution points for contraceptives. The merits of distributing contraceptives in schools need to be explored further, in addition to distribution through youth-friendly spaces in health facilities. Generating demand for contraceptives and sexual and reproductive health services, as described in the 2017–2021 Zambia Adolescent Health Strategic Plan, is essential for increasing contraceptive use in this age group. Increasing the use of modern contraceptives is essential and has been shown to have a significant impact on fertility, and maternal, new-born, and child survival. Contraceptive use can significantly reduce unintended pregnancies, abortions, and births, as well as avert thousands of child and new-born deaths, including hundreds of maternal deaths, annually [35].

## Conclusion

The findings of this paper show that contraceptive use among adolescent girls in Zambia has remained low over time, with only a modest increase from 8 to 11% between 1996 and 2013/14, which is much lower than the change in the general population. Over time, contraceptive use remained consistently low among younger, uneducated and unmarried sexually active adolescent girls, who comprise some of the disadvantaged sub-groups. In addition, whereas initial significant urban-rural differences in contraceptive use among adolescent girls did not occur in subsequent surveys, such differences began to emerge again in 2013/14. The findings suggest the need for targeted interventions to improve contraceptive use among sexually active adolescent girls in the country in general, and those who are disadvantaged in particular.

## Limitations

The study had some inherent limitations and strengths. The study was based on DHS data from four surveys. Some variables included in the analysis have either changed or been added over time. Furthermore, DHS data is based on self-reporting, and there may be social desirability biases in some responses. In addition, given the cross-sectional nature of DHS, it is not possible to make causal inferences about the relationships observed in the data. Despite the limitations, the study highlights patterns in contraceptive use among adolescent girls in Zambia, which have important implications for programs aimed at improving reproductive health outcomes among this sub-group of the population. The study is also based on a nationally representative sample of adolescent girls, which allows for generalizing the findings to all adolescent girls in the country.

## Data Availability

The data used in this paper are publicly available from the Demographic and Health Surveys (DHS) Program. Data can be accessed through their website https://dhsprogram.com/data/

## Abbreviations

AOR: Adjusted odds ratio
CI: Confidence interval
CPH: Census of population and housing
DHS: Demographic and health survey
LICs: Low income countries
UKZNBREC: University of KwaZulu natal biomedical research ethics committee
WHO: World health organization
ZDHS: Zambia demographic and health survey
